# Vaccine responses in newborns

**DOI:** 10.1007/s00281-017-0654-9

**Published:** 2017-11-09

**Authors:** Anja Saso, Beate Kampmann

**Affiliations:** 1 Centre of International Child Health, Department of Paediatrics, Imperial College London, W2 1NY, London, UK; 2 Vaccines and Immunity Theme, MRC Unit The Gambia, Fajara, The Gambia

**Keywords:** Neonate, Vaccination, Immune system, Infant, Immunisation, Immunity

## Abstract

Immunisation of the newborn represents a key global strategy in overcoming morbidity and mortality due to infection in early life. Potential limitations, however, include poor immunogenicity, safety concerns and the development of tolerogenicity or hypo-responsiveness to either the same antigen and/or concomitant antigens administered at birth or in the subsequent months. Furthermore, the neonatal immunological milieu is polarised towards Th2-type immunity with dampening of Th1-type responses and impaired humoral immunity, resulting in qualitatively and quantitatively poorer antibody responses compared to older infants. Innate immunity also shows functional deficiency in antigen-presenting cells: the expression and signalling of Toll-like receptors undergo maturational changes associated with distinct functional responses. Nevertheless, the effectiveness of BCG, hepatitis B and oral polio vaccines, the only immunisations currently in use in the neonatal period, is proof of concept that vaccines can be successfully administered to the newborn via different routes of delivery to induce a range of protective mechanisms for three different diseases. In this review paper, we discuss the rationale for and challenges to neonatal immunisation, summarising progress made in the field, including lessons learnt from newborn vaccines in the pipeline. Furthermore, we explore important maternal, infant and environmental co-factors that may impede the success of current and future neonatal immunisation strategies. A variety of approaches have been proposed to overcome the inherent regulatory constraints of the newborn innate and adaptive immune system, including alternative routes of delivery, novel vaccine configurations, improved innate receptor agonists and optimised antigen-adjuvant combinations. Crucially, a dual strategy may be employed whereby immunisation at birth is used to prime the immune system in order to improve immunogenicity to subsequent homologous or heterologous boosters in later infancy. Similarly, potent non-specific immunomodulatory effects may be elicited when challenged with unrelated antigens, with the potential to reduce the overall risk of infection and allergic disease in early life.

## Introduction

The World Health Organisation (WHO) estimates that 45% of deaths among children under the age of 5 years occur during the newborn period [1]. More specifically, neonatal infections currently account for ~ 700,000 of these deaths and ~ 7 million cases per year, with the greatest proportion affected and most severe outcomes in poorly resourced areas [2]. The burden of disease is high at this early stage due to the unique nature of the neonatal immune system specifically adapted to postnatal life, but simultaneously susceptible to infection and suboptimal vaccine responses. The transition from the sheltered in-utero environment to the ‘outside world’, the lack of defence from vaccine-induced antibody and the profile of early pathogenic organisms all contribute to the newborn’s vulnerability to microbial and environmental insults. Despite the limited ability of the neonatal immune system to develop potent memory responses, the success of the three vaccines administered in the immediate neonatal period, Bacillus Calmette–Guérin (BCG), hepatitis B vaccine (HBV) and oral polio vaccine (OPV), confirms that newborn vaccination can be effective at preventing three quite different diseases [3]. Furthermore, recent technological advances have enabled in vitro and in vivo modelling of early immune ontogeny with detailed characterisation of mechanistic processes. Along with the introduction of several significant global policy and funding initiatives to promote newborn and infant health, this has resulted in renewed interest in neonatal immunisation as an important tool to reduce the unacceptably high figure of neonatal mortality [4].

### The rationale for newborn vaccination

Neonatal immunisation would provide early protection for newborns and infants, narrowing the critical period of vulnerability intrinsic to routine vaccination schedules that start later in life. Other immunological advantages have also been hypothesised: fewer vaccine doses may be required if an immunogenic response is elicited at this early stage; there may be a general immunomodulatory effect, boosting immunity from birth before exposure to viral or bacterial pathogens [3, 5, 6]. Equally, neonatal vaccination is easily implementable, given that birth is a crucial point of contact with healthcare systems globally; as such, effective newborn vaccines would achieve high population penetration, particularly important in poorly resourced areas with otherwise limited health care services [6].

### The ideal neonatal vaccine

Important concepts for the successful development and impact of a neonatal vaccine include safety, immunogenicity and efficacy in addition to the establishment of a balance between reactogenicity/autoimmunity and immune tolerance [7, 8]. Ideally, a vaccine would be administered at birth (or before 4 weeks of age), via the oral rather than the intramuscular or subcutaneous routes, safely eliciting a strongly protective response after a single dose with minimal interference from maternal antibodies [3]. This response would be sustained or easily boosted as part of the subsequent routine infant immunisation schedule, without developing hypo-responsiveness when challenged with the same or concomitant vaccine antigens.

In this review article, we will discuss key features of the three vaccines currently recommended for use at birth. Furthermore, we will assess the main maternal, infant and environmental factors that may affect and/or hinder the success of future neonatal vaccines. Finally, we will summarise new strategies in the pipeline and remaining challenges to be addressed in the future.

## Systemic challenges to the use of vaccines in the neonatal period

### Immunogenicity

The main challenge to successful immunisation of newborns is achieving sufficient immunogenicity in the context of developing innate and adaptive immunity, primarily due to the distinct nature of neonatal leukocytes [2, 9, 10]. Crucially, the neonatal immunological milieu is skewed towards Th2-type immune responses, with dampening of Th1-type immunity and inflammasome pathways [4, 11–13]. These adaptations prevent alloimmune reactions between the mother and fetus, enable microbial colonisation and avoid excess proinflammatory responses [6, 10, 14]. Conversely, they render the newborn susceptible to infection and limit optimal vaccine responses [15, 16].

Neonatal CD4+ T cells are primarily recent thymic emigrants shown to have reduced immune function [17, 18]. Preferential CD4 T cell differentiation into Th2 effectors is thought to be partly mediated by epigenetic processes, specifically hypermethylation of IFNγ locus and hypomethylation of the Th2 locus [19]. This is further enhanced by the reduced capacity of antigen-presenting cells (APCs) to produce Th2-polarising cytokines, notably IL12 [2, 20]. Th1 cells also express IL4Rα/IL13Rα heteromeric receptors, with high risk of subsequent apoptosis on ligation with IL4, a product of Th2 cells [21]. Moreover, there is a qualitative and quantitative increase in suppressive T regulatory cells which secrete high concentrations of IL10 and TGF*β*, promoting tolerogenicity of APCs and limiting effector T cell responses [5, 22, 23].

In addition to these classic Th1/Th2 responses, Th17 proinflammatory responses might also contribute to an important component of vaccine-induced memory [2]. Production of Th17 cell promoting cytokines IL6 and IL23 dominates in term infants [24].

Humoral immunity in neonates is impaired with a subsequent need for repeated vaccine doses; lower antibody titres are secreted with a narrower antibody repertoire and suboptimal functional responses to some antigens when compared to older infants [3, 10, 25]. This reflects defects in neonatal B cell differentiation, with dominance of memory B cells versus plasma cells (PC), resulting in limited extrafollicular (EF) and germinal cell (GC)-derived plasma cell responses. This can be explained by both B cell-intrinsic and B cell-extrinsic factors: the former includes weaker B cell receptor-mediated signalling in naïve B cells, the lower expression of co-stimulatory molecules for T cells and the slow maturation of marginal zone B cells that recognise pathogen-associated molecular patterns (PAMPs) with subsequently reduced T-independent responses [25]. A key B cell extrinsic factor is the limited help provided by follicular dendritic cells (FDC) and/or T follicular helper (TFH) cells (the latter is mediated by IL12 which is reduced), thereby delaying GC induction [26–28]; dendritic cell activation signals and survival factors, such as A Proliferation Inducing Ligand (APRIL), are also suppressed [2, 27, 29]. On the other hand, high concentrations of adenosine in neonatal plasma selectively inhibit production of Th1-polarising cytokines via intracellular mechanisms, including induction of cyclic adenosine monophosphate (cAMP) [30, 31].

Given the limited exposure to antigens in utero, neonates are particularly dependent on their innate immunity to prime adaptive immune responses [32]. The unique characteristics of the newborn’s innate immunity are therefore critical in explaining why they have suboptimal vaccine responses in comparison to older infants [5, 33]. Above all, the functional activity of neonatal APCs is impaired, primarily because they express pattern recognition receptors (PRR), such as Toll-like receptors (TLRs), with distinct functional responses including limited antiviral and Th1-polarising cytokine production to most stimuli [2, 34, 35]. This cytokine ontogeny is thought to be due to epigenetic changes as well as differences in activity of interferon response factor (IRF) transcription factors. For example, there is impaired nuclear translocation of IRF7 thereby inhibiting transcription of IFNα and IFNβ; reduced IRF3 DNA binding activity and association with the co-activator CRB-binding protein (CBP) also lead to lower type 1 IFN production [24]. However, the precise molecular mechanisms underlying TLR-mediated innate function in early life have not been fully delineated and require further attention, since their potentiation would help to induce Th1 and TFH cell responses, thereby overcoming newborn adaptive immune deficiencies [5].

### All vaccines are not equal

The impact of immunisation on this unique neonatal immune regulation milieu may vary between vaccine types. Infants immunised at birth with HBV or OPV develop lower Th1 type responses than adults. However, such Th1 responses are associated with higher antibody responses than in adults receiving booster doses [36, 37]. By contrast, it is now well established that BCG induces potent mature Th1 responses in newborns [38].

Multiple studies have explored the variable capacity of different vaccines to induce appropriate Th1 responses at birth, identifying determining factors such as antigen dose, type of adjuvant and route of administration [11, 13, 15]. Above all, the ability of APCs to potentiate adaptive immune responses plays a crucial role [5].

### Immune interference and hypo-responsiveness

There is theoretical concern that neonatal vaccination may result in the subsequent development of immune hypo-responsiveness, particularly when challenged with either the same antigen (‘immune paralysis/tolerance’) and/or concomitant antigens (‘vaccine interference’) in subsequent infant immunisation schedules [3, 6, 39].

Halasa et al. demonstrated that the administration of an additional dose of diphtheria, tetanus, acellular pertussis (DTaP) at birth was safe but also associated with significantly reduced antibody response to three of the four pertussis antigens (pertussis toxin and pertactin at 6, 7 and 18 months and fimbrae at 6, 7, 17 and 18 months) and to diphtheria antigens at 7 months when compared with controls. Immune responses to all other vaccine antigens were comparable including HBV and tetanus toxoid [40].

By contrast, Knuf et al. showed that priming at birth with a three-component standalone aP vaccine was well tolerated and resulted in significantly higher antibody responses to the three pertussis antigens at 3 months of age (after the first dose of DTaP vaccine had been given) in comparison to controls. Early neonatal immunisation with aP did not elicit immunologic tolerance to pertussis antigens, with all subjects reaching antibody concentrations above the protective threshold 1 month after completing the vaccine course, although it did dampen responses to *Haemophilus influenza B* (Hib) and HBV vaccines [41]. Given that the maternal antibody levels in newborns were comparable in both studies, their contrasting results were thought to be secondary to the different vaccine formulations and/or adjuvants used or to the additional birth dose of HBV given in the former trial. Furthermore, Knuf et al. have proposed that the robust secondary T cell pertussis-specific responses, induced after the first DtaP-IPV-HBV/Hib vaccine dose, may have subsequently interfered with adequate recruitment of CD4+ T cells needed to activate a strong primary response to concomitantly administered HiB and HBV antigens, known as ‘bystander interference’ [41, 42].

Other studies using different vaccines have disputed this concept of immune downregulation. As part of the large randomised, multi-centre clinical trial of BCG given at birth, the Danish Calmette Study, Nissen et al. investigated the effect of BCG at birth on the antibody response to the three routine vaccines against DTaP-IPV-Hib and Prevenar 13 in a subgroup of participants. BCG did not reduce the probability of having antibody titres above the limit of protection with seroprotective levels detected for each of the three vaccinations in almost all children 4 weeks post vaccination. There was also no difference between BCG and controls with respect to geometric mean concentrations (GMC) of antibody even when adjusted for the background factors [43]. Similarly, no interference has been observed between OPV and the vaccines administered alongside it as part of the WHO routine immunisation schedule [44].

## What can we learn from vaccines currently administered to neonates?

### Bacillus Calmette–Guérin

BCG is a live attenuated *Mycobacterium bovis* vaccine administered within the first few days of life in most countries to prevent tuberculous (TB) meningitis and disseminated TB disease in children. It is the most widely used vaccine worldwide with an overall excellent safety record. Adverse reactions range from localised inflammation and scarring at the site of inoculation (95% of healthy neonates) to more serious, systemic events including axillary and cervical lymphadenopathy and, very rarely (< 1 in million), disseminated BCG infection [45, 46].

As yet, no surrogate immune marker of BCG-induced protection has been identified [47]. Clinical markers of its efficacy are used instead. Crucially, there is 73% protective efficacy against TB meningitis and 77% against miliary TB [45]. However, there is no convincing protection against primary pulmonary infection or reactivation of latent TB, and protective efficacy declines to non-significant levels after 10–20 years [45, 48, 49]. Moreover, BCG efficacy is variable and may depend on several factors including the immune status of the recipient [50], prior exposure to environmental mycobacteria (both *M. tuberculosis* and non-tuberculous mycobacteria) [51], strain variation in BCG preparations [52] although this has been contested [53], genetic or nutritional variability and environmental factors including sunlight exposure and poor cold-chain maintenance [6, 51]. Prior exposure is thought to both limit replication of BCG and/or confer protection equivalent to BCG (‘masking’). Given that this is dependent on age, it would explain why mycobacteria-naïve newborns appear to benefit more from BCG vaccination than older individuals [53].

BCG does not contain any exogenous adjuvant but is inherently ‘self-adjuvanted’ because *Mycobacteria* induce immune responses via TLR2, TLR4 and TLR8 [52]. BCG induces a potent Th1 polarised immune response to mycobacterial antigens, thereby overcoming the common obstacle faced by newborn vaccinations and confirming that reduced Th1 capacity in neonates is not absolute [54]. Studies among infants demonstrate BCG-associated induction of CD4+ and (to a lesser extent) CD8+ T cells, IFNγ, IL2, TNFα and polyfunctional CD4+ T cells [55]. However none of these responses were found to correlate with protection against TB in BCG-immunised infants [56].

Neonatal BCG vaccination may also have non-specific, heterologous beneficial effects, boosting the innate and adaptive immune responses to unrelated antigens and reducing morbidity and mortality from non-tuberculous infections as well as allergic diseases [57–62]. The nature and significance of such effects, however, remains controversial and it cannot be excluded that neonatal vaccination may have a similar masking effect on vaccines administered later in infancy, as already observed between early exposure to (environmental) mycobacteria and BCG [63–66].

Ota et al. demonstrated that BCG boosted cytokine and antibody responses to unrelated vaccine antigens (HBV and OPV) in neonates, probably by influencing maturation of dendritic cells. The finding that both Th1 and Th2 cytokines increased suggests that the magnitude but not the quality of neonatal responses to vaccines was influenced by BCG [38]. Similarly, an Australian study found that BCG was associated with significantly higher response of several subclasses of the pneumococcus conjugate vaccine (PCV) routinely given during childhood [67].

Although the underlying immunological mechanisms have not been fully elucidated, two hypotheses have been proposed for these non-specific effects: ‘trained innate immunity’ and ‘heterologous immunity’. ‘Trained innate immunity’ describes the ability of the innate system to generate immunological memory and therefore be ‘trained’ to provide partial protection against subsequent infections, independent of classical T and B cell adaptive immunity [68–70]. Natural killer (NK) cells and monocytes have emerged as central to trained immunity in mammals via functional reprogramming of PRR (e.g. NOD2 receptor) [71, 72]. Primary exposure to microbial ligands alters the fate of monocytes, dependent on the nature and concentration of the ligand, mediated by epigenetic mechanisms at the level of H3K4 trimethylation [71].

The second immunomodulatory process proposed is ‘heterologous immunity’. This describes the effects on the adaptive immune system, primarily T cell-mediated cross reactivity between vaccine-related and vaccine-unrelated antigens. As such, T cell vaccine responses may be impacted and modified by encounters with pathogens from previous infections [71, 73, 74]. Although the WHO recently recommended that the existing evidence was insufficient to prompt a revision of immunisation policy, further studies are required to characterise the magnitude, duration and mechanisms of the non-specific effects of vaccines and, therefore, their potential implications for infant health [65, 66].

### Hepatitis B vaccine

There is extensive evidence of the excellent safety and immunogenicity profile of hepatitis B vaccines. Administration of HBV, in some cases with hepatitis B immunoglobulin (HBIG), remains the most effective way of preventing the high risk of mother-to-infant transmission of HBV [6, 75–77], and it has been demonstrated that timely administration of the birth dose results in the highest vaccine effectiveness.

The current widely used r-HBsAg vaccines, available since 1986, are a viral subunit that has been transfected with a plasmid that contains the *S* gene (codes for HBsAg) either as a single preparation or in combined form. Alum, a chemical compound containing aluminium salts, is added as adjuvant.

The vaccine is highly immunogenic and induces potent neutralising antibody following a series of at least three doses. Recommended vaccination schedules that include a birth dose will prevent most perinatally acquired infections and offer early protection from horizontal transmission.

Anti-HBs antibodies are a marker of immunity and a titre of anti-HBs antibodies to HBVsAg ≥ 10 IU/L indicates seroprotection if measured 1–3 months after completion of the primary immunisation series. In healthy infants, 30–40% protection against HBV infection is achieved with one dose of the vaccine, 50–75% protection with two doses and > 90% with three doses [6].

Four intramuscular vaccine doses are recommended in susceptible infants over the first year of life in the UK (three in the USA). Side effects are generally mild including pain at the injection site (3–29%), mild fever > 37.7 °C (1–6%), malaise, headache, arthralgia and myalgia [6]. HBV immunisation induces at least equivalent antibody responses in newborns and adults; this suggests that the capacity of the newborn to develop antibody responses depends on the nature of the immune stimulus [9, 36]. Moreover, the success of the HBV schedule confirms that vaccination at birth can elicit potent memory B cell responses that promote immunogenicity of subsequent vaccine booster doses, irrespective of primary antibody response [5, 44].

Long-term protection against HBV infection depends on the persistence of strong immunological memory and can vary between individuals. After primary immunisation with HBV vaccine, anti-HBs concentrations wane quite rapidly within the first year and more slowly thereafter. However, even if anti-HBs concentrations decline to below 10 IU/L, immune memory continues to persist over a longer time period. Booster doses are not currently recommended for fully vaccinated, immunocompetent individuals.

Similar to the immune response to HBV infection, T cell dependence of the immune response to hepatitis B vaccination has been demonstrated, and it was shown that non-responding infants had a reduced capacity to adequately expand and differentiate TH cells.

The T cell responses elicited by HBV have been shown to vary between newborns and adults. A study by Ota et al. demonstrated lower interferon-γ production (reflective of Th1 immunity) but higher Th2 memory responses in those vaccinated at birth when compared with adults [36].

### Oral polio vaccine

Two types of polio vaccines are in use worldwide: intramuscular (or subcutaneous) inactivated polio vaccine (IPV) and orally administered, live attenuated polio vaccine (OPV). In polio-endemic countries and in areas at high risk for importation and subsequent spread, WHO also recommends an OPV dose at birth (called ‘zero’ dose), followed by the primary series of three OPV doses with at least one IPV dose [78]. OPV therefore remains the first mucosal vaccine received by most newborns in low- to middle-income countries. A trivalent OPV formulation (tOPV) was used worldwide until April 2016 when it was replaced with bivalent type 1 and type 3 OPV (bOPV) during a global synchronised switch. Withdrawal of type 2 OPV was required because type 2 wild polioviruses no longer circulate, and its continued use had been responsible for a disproportionate number of vaccine-associated paralytic polio (VAPP) and circulating vaccine-derived polioviruses (cVDVP) cases [78]. There is no extrinsic adjuvant within the OPV formulation; instead, it contains single-stranded RNA which activates the innate immune system by stimulating TLR8 [79].

Halsey et al. comprehensively summarised a large dataset obtained from different countries, using serological assays where results were adjusted for estimated decline of maternal antibody, and viral excretion assays in stool [44]. Overall, the summary data demonstrate that when administering a dose of tOPV and DTP in the first week of life, 50–100% of newborns will benefit by developing active intestinal infections and local immune responses, while 30–70% of neonates develop serum antibodies to one or more poliovirus types. The wide confidence intervals are explained by a number of factors such as persisting maternal antibody at time of vaccination, breastfeeding, varying intervals of administration after birth and a range of geographic settings. Based on these data, the authors recommended the use of tOPV at birth in high risk areas where poliomyelitis has not been controlled [44].

A subsequent Egyptian trial confirmed the immunogenicity of a monovalent type 1 OPV given at birth and its superiority over previous trivalent formulations. Fifty-five percent of newborns in the monovalent-vaccine group seroconverted 30 days after priming. Nearly half of infants with high maternal antibody titres still secreted antibodies against type 1 poliovirus. Following challenge with type 1 OPV at 1 month of age, 75% of infants did not excrete type 1 IPV [80].

By contrast, a study in Belgium used OPV as a model of early immunisation to investigate the capacity of young infants to develop cellular immune responses. They showed that weak IFNγ and cell-mediated responses (i.e. limited Th1 responses) to polio antigens were induced post neonatal OPV when compared to immune adults. However, the titre of neutralising antibodies was high, above the protective threshold, similar to previous studies [37].

In summary, the considerable experiences with existing vaccines have shown that newborns are in fact able to mount robust immune responses to neonatal vaccines, particularly to live attenuated vaccines, but that such responses are influenced by a number of co-factors, as discussed in later sections.

## What can we learn from vaccines that have been investigated for use in neonates but are not currently part of recommended regimens?

### Pneumococcal vaccines (PCV)

Two recent trials have demonstrated that the delivery of seven valent pneumococcal conjugate vaccine (PCV7) to neonates may be safe and immunogenic, with no evidence of hampering potential long-term protection or inducing immune tolerance. The first trial, undertaken in Kenya, randomised young infants to either receive seven-valent PCV at 6, 10 and 14 weeks (EPI group) or 0, 10 and 14 weeks (neonatal group). Serotype-specific serum IgG and avidity were measured at birth and 6, 10, 14, 18, 36 and 37 weeks. Infants were then boosted with either seven-valent PCV or one fifth dose of pneumococcal polysaccharide vaccine at 36 weeks. Results demonstrated excellent safety with minimal adverse effects. At both 18 and 36 weeks, there was no significant difference in the proportion of IgG above the protective threshold against each serotype between infant groups. However, geometric mean concentrations (GMCs) were lower in the neonatal group for serotypes 4, 9V, 18C and 19F at 18 weeks and for serotype 4 at 36 weeks. By contrast, avidity was significantly higher in the neonatal compared to the control group for serotypes 4, 6B and 19F at 18 weeks and for serotype 19F at 36 weeks likely because affinity maturation would have occurred for longer since birth. The clinical significance of these avidity findings is uncertain and it could be argued that lower GMC could lead to non-protective responses at an earlier stage. However, this did not occur during the follow-up period of 37 weeks. Response to the 36-week boosters and prevalence of vaccine-type/non vaccine-type carriage were comparable between groups, suggesting absence of immunological tolerance after neonatal vaccination [81, 82].

Similarly, Pomat et al. conducted an open randomised trial in Papua New Guinea to compare infants given PCV7 in a 0-1-2-month (neonatal) schedule with that of the routine 1-2-3-month (infant) schedule. All infants received 23-valent pneumococcal polysaccharide vaccine (PPV) at age 9 months. By 2 months of age, GMCs for serotypes 4, 9V, 18C and 19F were significantly higher in the neonatal than in the infant group, taking into account that the neonatal group had received two doses of PCV7 compared with the single dose in the infant schedule. Post-hoc analysis of the differences in proportions of children with protective antibody levels at age 2 months did not demonstrate superiority of two doses in the neonatal group over one dose in the infant group. While all antibody responses to vaccine types in the neonatal group were non-inferior to those in the infant group at age 2 months, over time the infant-immunised group generally had higher Ab levels than the neonatal-immunised group. On subsequent challenge with PPV at 9 months, significantly higher serotype-specific IgG concentrations above the protective threshold were induced in PCV7 primed compared to unprimed infants; neonatal and infant groups had equivalent antibody levels, which persisted until the end of follow-up at age 18 months. These results again suggest an absence of tolerance following neonatal PCV vaccination [82, 83].

### Pertussis vaccines

Immunisation within 24 h of life with whole cell pertussis or combined with diphtheria and tetanus vaccines has previously demonstrated a good safety profile, with no moderate or major adverse effects documented [84]. Th1-type immunity was preferentially induced, in a similar way to BCG. However, the serological response was suboptimal and a reduced response to pertussis boosters was documented in 75% of study subjects until 5 months of age, independent of maternal antibody titre which was low. This can probably be attributed to antigen-specific immunological hypo-responsiveness, already discussed previously. By contrast, if immunisation was delayed to 3 weeks of age, the serological response was adequate [85, 86]. Based on these findings, the WHO recommends starting pertussis immunisation schedules from 6 weeks of age.

Due to the recent resurgence of severe pertussis in early life, there has been renewed interest in administering aP vaccines at birth, either as a sole vaccine or as part of DTaP combined vaccine. The results of these studies have been conflicting, as discussed previously, with concerns regarding tolerogenicity both to the same antigen administered later in infancy or ‘bystander interference’ to concomitant antigens. [40, 41, 87, 88]

### Vaccines against viral pathogens

The high burden of respiratory and gastrointestinal disease in early life warrants the further study and development of neonatal vaccines against RSV, parainfluenza, influenza and rotaviruses, ideally administered via the oral or nasal route to induce effective mucosal immunity. Neonatal immunisation against RSV is currently under consideration with several vaccine candidates under development, but none has yet progressed to licencing [89].

A recent pilot study enrolling 66 infants into two equally sized groups comparing pentavalent rotavirus vaccine (RV5) administered on an early alternative dosing schedule (at 2–5 weeks of age) compared with RV5 administered on the current WHO standard schedule, measuring safety, serum-neutralising antibody (SNA) and IgA geometric mean titers (GMTs). RV5 was found to be generally well tolerated and immunogenic in the neonatal period, although the rotavirus SNA serotype 4 response was less robust in newborns than in infants on the standard schedule [90].

## Co-factors affecting responses to newborn vaccines

### Maternal infections and maternal antibody

The maternal factors potentially affecting the success of neonatal immunisation are threefold: maternal-fetal transfer of antibodies, maternal-fetal transfer of pathogenic organisms and chronic maternal infections.

Maternal antibodies, transplacental or milk-derived, may potentially interfere with the neonatal adaptive immune response and subsequent vaccine responses [91, 92]. This has already been shown in the context of maternally derived antibodies inhibiting immunogenicity of the measles vaccine in infants [93]. The suppressive effect is thought to depend on the ratio between antibody titre and vaccine antigen dose, given that circulating antibodies hide the antigen from the host’s immune cells thereby masking B cell epitopes [92]. This is exacerbated by the unique nature of the neonatal Fc receptor which increases the serum half-life of passively transferred antibodies [5, 94]. By contrast, other studies have shown that the infant’s APC uptake and T cell responses appear to be largely unaffected by maternal antibody [13]. Further research is currently ongoing to gain better mechanistic insights. Antibodies in breast milk may similarly limit the efficacy of oral vaccines at the mucosal surface, although this has been contested [95–97]. A recent study by Ali et al. demonstrated that withholding breastfeeding around the time of rotavirus vaccine administration did not improve vaccine immunogenicity with no increase in anti-rotavirus IgA seroconversion measured [98].

Transmission of pathogens across the placenta to the infant can also play a role in early life immunity with a potential impact on newborn vaccine responses: HIV-associated immune compromise, particularly cellular immune deficiency, exacerbates poor neonatal vaccine outcomes and some adverse effects. In a study carried out in South Africa between 2004 and 2006, the pooled incidence of disseminated BCG was estimated to be as high as 992 per 100,000 HIV-infected vaccinees, almost 1000 times higher than those who were HIV-uninfected [99]. The mortality rate associated with disseminated BCG disease was > 70% [99, 100]. Other pathogens that are vertically transmitted and may potentially affect neonatal immunity include toxoplasmosis, rubella, CMV and other herpes viruses, syphilis, enterovirus, parvovirus B19 and hepatitis viruses. A comprehensive discussion of the impact of each disease on vaccine responses is beyond the scope of this review.

Chronic maternal infections may shape neonatal vaccine responses by their impact on maternal antibody transfer across the placenta or their generalised effect on the developing neonatal immune system. This occurs independently of vertical transfer of pathogenic organisms and is secondary to exposure to microbial antigens, either free or complexed with immunoglobulins crossing the placenta. Subsequently, there is sensitisation in-utero and induction of adaptive, inflammatory and regulatory responses [101]. The unique maternal disease profile during pregnancy may contribute to the observed differences in immune responses of infants in developed compared to developing countries [102].

During pregnancy, prevalence of infection with at least one helminth species is 10–70% in endemic regions [101]. Detection of specific immunoglobulins, particularly IgE, to various helminth species in cord blood reflects in-utero sensitisation of both B and T lymphocytes to microbial antigens, while parasites are rarely transmitted vertically [103, 104]. Poor T cell responses to tetanus toxoid (TT) immunisation and reduced HiB vaccine-specific antibody responses have been reported in neonates born to mothers with lymphatic filariasis and/or schistosomiasis [105]. Furthermore, Malhotra et al. found that Kenyan infants born to mothers infected with *Schistosoma haematobium* and sensitised in utero had lower IFNγ responses to BCG than did unexposed infants [106]. A similar pattern was seen in the context of maternal filariasis during pregnancy [106]. By contrast, Elliot et al. concluded that maternal helminth infection may have little, if any, adverse effects on the outcome of infant immunisation. Of note, only maternal *Mansonella perstans* infection was associated with significantly higher IL10 responses to BCG and tetanus immunisation but with no reduction in IFNγ, IL5 and IL13 responses [107].

Maternal infection with *Trypanosoma cruzi* and congenital Chagas disease induces a trend to type 1 polarisation of infant immune responses to vaccines. High levels of IFNγ were noted in response to BCG administered in exposed but uninfected infants compared with congenitally infected infants or unexposed and uninfected infants [108].

Data on vaccine responses of HIV exposed but uninfected newborns are conflicting. On one hand, studies have found that their humoral and cell-mediated responses to vaccines, including to the measles vaccine and BCG, are similar to responses in unexposed infants [109, 110]. Moreover, Jones et al. demonstrated that HIV exposed uninfected infants born in South Africa mounted higher increments of antibody responses to pertussis and pneumococcus vaccines [111]. This may be because the reduced transfer of specific maternal antibodies noted in this cohort mitigated the poor vaccine-induced humoral responses seen in early infancy [33]. The same research group also studied the impact of maternal HIV infection and demonstrated that HIV exposed, uninfected infants have normal responses to BCG vaccination administered at birth. T cell subsets are affected, however, which likely reflects priming of the developing immune system even in the absence of infection. Similarly, sensitisation to mycobacteria resulted in a positive correlation between maternal and newborn levels of BCG-specific CD4 T cells. This again suggests that maternal cellular responses may shape the infant response to BCG antigen. Following administration of BCG at 6 weeks of age, however, no difference in subsequent BCG-specific T cell proliferative responses or cytokine induction was documented at 16 weeks of age between those infants with either exposure to maternal HIV and/or latent TB [112]. Therefore, any effects of maternal HIV and possible TB infection on infant immune profiles at birth are considered transient, as long as the infant remains uninfected. Given this, HIV exposed, uninfected infants are equally likely to respond to and be protected by BCG vaccination as HIV unexposed infants [33, 112]. Not all studies, however, currently agree on this conclusion. Data from two Brazilian studies suggests that HIV exposed but uninfected infants have altered T cell responses to BCG with lower TT- and HBV-specific antibody levels [113, 114].

Finally, placental malaria, secondary to maternal infection with *Plasmodium falciparum* or *Plasmodium vivax*, may impact neonatal immunity and vaccine responses. Above all, placental malaria is associated with reduced transfer of specific maternal antibodies, including RSV, varicella zoster, herpes simplex and measles and TT [115–117]. This may be due to pathological changes in the placental structure in endemic settings, with subsequent damage to Fc receptors [89, 115–117]. Furthermore, similar antibody and T lymphocyte responses to TT were noted in infants born to mothers with or without placental malaria [118]. Ultimately, further work is required to elucidate these effects. The mechanisms may be similar to those proposed in maternal HIV, in that the limited transfer of maternal antibody may overcome potential blunting of neonatal humoral responses to early vaccination.

### Neonatal co-factors

In addition to maternal factors, genetic variation among hosts plays a key role in the observed heterogeneity of neonatal and infant vaccine responses [119]. Marchant et al. measured antibody responses to tetanus toxoid, measles and total IgG in 210 Gambian twin pairs, estimating the environmental versus genetic components of any variation documented. They found that genetic determinants control the early phase of the vaccine antibody response, while the environment predominantly influences antibody persistence and avidity maturation [120]. More recently, further studies have applied genome-wide association studies in order to attempt to identify the genetic determinants of different vaccine responses against hepatitis B, measles and rubella [121].

The quantity and quality of innate and adaptive responses to newborn immunisation are further impaired in babies born prematurely. As such, the concerns regarding immunogenicity and safety are even more marked, particularly given the lack of detailed immunological studies in this age group, mainly limited by the small amounts of blood available [122, 123]. For a number of antigens, the humoral response to initial vaccine doses may be lower than that of term infants, but the protective threshold is often reached and memory successfully induced, sometimes with an additional dose to achieve persistence of protection [123]. Again, the lower titres of maternal antibodies seen in prematurity may enhance this effect. On the other hand, it is widely accepted that HBV is less immunogenic in preterm infants with birth weights < 2 kg than in term infants [124, 125]. Nevertheless, unless they have underlying medical complications, these infants respond to HBV by 30 days of age regardless of birth weight or gestational age [124].

Baxter et al. concluded that immunogenicity in this cohort is vaccine specific: robust responses are seen following TT and inactive preparations; however, subunit formulations are less optimal. As such, they recommend administering additional booster doses and/or serology testing for infants < 32 weeks [122]. Further research in this area would give important insights into the ontogeny of the maturing fetal and neonatal immune system and should be encouraged, given that novel tools are now available potentially applicable to very small volume samples.

In parallel to the significant impact of maternal co-infections and maternal-fetal disease transmission on neonatal immune responses, other co-morbidities in the newborn may have equally profound effects. One example is congenital non-infective immunodeficiency such as severe combined immunodeficiency disease (SCID). BCG vaccine has a very high rate of complications in SCID patients, as in HIV disease, with increased vaccine-associated morbidity and mortality rates [126]. Disseminated BCG infection in children is also linked to rare immunodeficiencies of the IFNγ and IL12 pathways [50]. Furthermore, acquired infections may be important. Few studies have elucidated these interactions in the neonatal context although more work has been done in older children. Infant malaria is associated with lower IFNγ, IL5 and IL13 (i.e. Th1) responses to both BCG and tetanus immunisation [107]. In vitro incubation of monocytes with *P. falciparum*-infected red blood cells has been reported to lead to enhanced responsiveness to TLR ligands, which may shape neonatal immune responses [127].

### Environmental

It is well established that neonatal immunity and therefore vaccine responses vary considerably across different geographical settings [33]. For example, Lalor et al. demonstrated that 3 months post-BCG immunisation, 100% of UK infants made an IFNγ response to *M.tb* purified protein derivative (PPD), compared to 53% of Malawian infants in whom responses varied by season of birth and possibly influenced by early exposure to environmental mycobacteria, as earlier discussed. Furthermore, there was a higher Th2- and lower Th1-type response to PPD in Malawian infants compared with UK infants [128]. Similarly, Hur et al. reported that both geographical location (Malawi, Gambia and UK) and season of birth (dry vs. wet) significantly affected the cytokine profile and immunogenicity observed in response to PPD in infants at 3 months post BCG vaccination [129]. However, the type of assay used meant that it was not possible to show which cells were primarily involved in mediating these differences and the underlying mechanisms remain unclear [33].

Finally, environmental factors combined with host genetics influence bacterial colonisation after birth and the establishment of gut microbiota, regarded as crucial for optimal host immune development. This is illustrated by the finding that germ-free mice are at increased risk for infectious as well as autoimmune disease [2, 130, 131] Currently, the impact of the microbiome on early vaccine responses is still poorly understood. In one study, TLR5-mediated sensing of the microbiota was found to drive lymph node plasma cell differentiation and enhance antibody responses to inactivated polio and influenza vaccines [132]. Moreover, it is already well known that immune responses to oral vaccines in developing countries are suboptimal, possibly related to gut dysbiosis. Huda et al. investigated if stool microbiota composition predicted infant oral (OPV) and parenteral (BCG, HBV, tetanus toxoid) vaccine responses at 6, 11 and 15 weeks of age in a small group of 48 Bangladeshi infants. They demonstrated that *bifidobacterium* predominance may enhance thymic development and responses to both oral and parenteral vaccines early in infancy. By contrast, greater bacterial diversity may cause systemic inflammation (neutrophilia) and lower vaccine responses [133]. There is now need for similar studies in the neonatal period.

## The future: novel approaches to neonatal immunisation

### Optimise research models

In order to develop improved neonatal vaccines, better and more applicable research models will be required; this would enable the accurate assessment of both vaccine immunogenicity and safety [3, 5, 6]. Neonatal trials, even pilot studies, are fraught with practical and ethical challenges. Nonetheless, it is not sufficient to predict the response of human newborns to vaccines from studies of older infants or adults. Equally, although animal models, particularly neonatal ones, can provide useful insight into early life immunity, they carry inherent disadvantages which may preclude the direct translation of results and conclusions to humans [6]. Some mammalian species, including horses, pigs and ruminants, differ in the type of placentation and relative placental and colostral transfer of immunoglobulins to the fetus and newborn [134]. Similarly, the innate immune system highly varies between species (e.g. murine vs. human). The neonatal period in mice typically refers to the first 7 days of life and therefore any experiments that exceed this time period may not be applicable to human newborns [5, 6, 134].

Novel approaches to neonatal immunisation are threefold: new types of vaccine configurations (varying both mechanism of action and antigen-adjuvant formulations), new types of vaccine delivery and new types of infant immunisation strategies. These approaches aim to overcome deficiencies in neonatal innate and adaptive immune responses and thereby enable effective newborn vaccination [5]. The dynamic ability of the early immune system to balance often opposing demands, genetically encoded to maintain immune homeostasis versus environmentally driven to challenge a range of potentially pathogenic organisms, demonstrates its powerful potential for plasticity [2].

### Novel vaccine configurations

The success of neonatal BCG and HBV emphasises that (1) sufficiently robust adaptive immune responses and functional, seroprotective antibody titres can be generated in the newborn period (2) this is will only occur in response to sufficiently potent antigen-adjuvant combinations and/or appropriate cellular interactions (3) ideally focus should be on potentiating innate immune signalling pathways, particularly recruiting and activating APCs, to help overcome adaptive immune deficiencies, including Th2-type skewing and dampened Th1-type immunity while boosting B cell responses [32, 135]. Finding a novel vaccine type is, therefore, primarily a search for optimal antigen-adjuvant combinations that will be both effective and safe at birth. Costly antigens and multiple vaccine doses may also be avoided [5].

Adjuvants boost infant immunity through multiple mechanisms, including the following: activating innate immune responses; increasing half-life of the vaccine antigen by creating a ‘depot effect’; assembling and directing antigens towards APCs, subsequently activating them; eliciting stronger mucosal responses; supporting cell-mediated immunity by enhancing cytotoxic or Th-1 type T cell function [136]. Currently alum is the only licenced adjuvant in neonates, despite studies suggesting that it does not promote robust Th1-type immunity [137] .

MF59, an oil-in-water emulsion recently being investigated for use in early life adjuvanticity, assembles antigen-containing microspheres and has been showed to have a good safety profile. In neonatal murine models, it both induces a ‘depot effect’, prolonging stimulation of the vaccine antigen, and enhances APC recruitment and activity, subsequently promoting CD4+ effector T cell activity and strong B and T cell memory responses [5]. Moreover, TFH cell function is boosted which mediates B cell adjuvanticity, with secretion of persistently high and broader range of antibody levels [29].

Another approach to promoting robust T cell responses, including shifting the polarisation to Th1-type immunity, is to tackle neonatal APC impairment by exogenously administering co-stimulatory signals. This is particularly important if endogenous cytokine secretion is deficient or transcription within dendritic cells (DCs) is repressed, such as with IL12 [138]. As discussed earlier, IL12 is critical in mediating DC-directed T cell differentiation to TFH as well as enhancing activity of cytotoxic and Th1-type T cells. Co-administration of IL12 and influenza subunit vaccine to newborn mice led to increased splenic expression of IFNγ, IL10 and IL15 mRNA and enhanced protective efficacy of antiviral immunisation [139]. By contrast, synergistic stimulation of DCs with at least two TLR agonists demonstrates enhanced endogenous Il-12p70 production in cord blood [140]. Given its function, effective induction of IL12 is therefore a priority in vaccine design.

Most importantly, new vaccine formulations are being investigated that boost innate immunity by judicious targeting of PRRs, primarily TLRs [79, 135]. Much interest has focused on specifically stimulating TLR3, TLR7, TLR8 and TLR9 receptors, which are located within endosomes and display robust responses to stimulation in neonates [5]. This is probably because they can utilise adenosine-refractory intracellular pathways [141]. CpG DNA, a TLR9 ligand, has been shown to increase pertussis toxoid-specific antibody secretion, along with immune defence peptides and polphasphazenes, following newborn and adult vaccination in mice [142]. Similarly, TLR8 agonists, such as certain synthetic imidazoguinolines and single-stranded viral RNA, are particularly effective at stimulating human neonatal APCs in vitro as well as eliciting TNF and IL12p40/70 secretion and promoting up-regulation of the co-stimulatory molecule CD40 [143]. Equally, these agonists may also mitigate the suppressive effects of T regulatory cells that induce APC tolerance and limit robust adaptive immune responses [144]. Moreover, TLR agonists mediate B cell adjuvanticity, with the production of high affinity B cell effectors, by acting on multiple innate cells including dendritic cells and enhancing TFH responses [2]. Most recently, Dowling et al. demonstrated that TLR8 agonist nanoparticles (polymersomes) mimic immunomodulatory mechanisms seen following BCG administration, for example newborn DC maturation profiles are similar to those induced by BCG but with greater IL12p70 secretion [145]. In particular, the ability to stimulate multiple TLRs simultaneously has a synergistic effect, with recent focus on combined TLR7/TLR8 stimulation to circumvent impairment of newborn APC responses. R848 (also known as imiquimod, a type of imidazogluinoline) ligates both TLR7 and TLR8 receptors simultaneously, resulting in more marked production of TNFα and IL-1β than if these sites are stimulated individually [141].

As we have seen, adjuvants in the neonatal context would primarily help to overcome functional peculiarities of neonatal APCs, thereby promoting both cell and humoral immunity. Further work is required to yield more effective, targeted interventions at PRR and particularly TLR 3/7/8/9 sites, while still monitoring for reactogenicity and potentially severe adverse effects, including development of toxicity, neurological reactions or autoimmune disease [5, 6, 136].

Finally, establishing a novel mechanism of action may be another potentially effective strategy. In particular, DNA-based immunisation has shown promise in the context of protecting newborn mice against malaria. Vaccinated neonates, including those born to immune mothers, were noted to mount CD8+ T cell-mediated protection similar to adults [146].

### Novel mechanisms of vaccine delivery

Administration of neonatal vaccinations by the mucosal route may be superior to parenteral immunisation. This is particularly true for RSV and rotavirus which are responsible for the high burden of infant respiratory and gastrointestinal diseases, respectively, worldwide. A study by Noh et al. using a murine model demonstrated that intranasal administration of RSV glycoprotein core fragment (Gcf) at birth can elicit systemic humoral immune responses and elevated IFNγ secretion, protecting newborn mice against RSV challenge without development of lung eosinophilia, even in the presence of high RSV-specific maternal antibody titres [146].

Intracytoplasmic delivery of antigens is another novel option being considered. Indeed robust neonatal adaptive responses are dependent on the entrance of vaccine antigens in to the cytoplasm of APCs. To overcome potential blunting from maternal antibodies, Chen et al. used a neonatal murine model in the context of influenza immunisation, to show that mothers and their offspring should be vaccinated with different vaccine types targeting distinct antigens. The application of an attenuated strain of the intracellular bacterium *Listeria monocytogenes* to deliver heterologous antigen directly and efficiently to APC cytoplasm may also be a promising vaccine vehicle for the newborn. A strong CD8+ and CD4+ Th1 type memory response was induced, even in the presence of pre-existing maternal immunity. Importantly, this approach seemed to be safe in neonatal mice but all remains untested in human infants, given the potentially harmful effects of this pathogen [147].

### Combination strategies

Recently, there has been an interest in using neonatal vaccines as primers (vs. single dose), in order to potentiate immune responses to homologous or heterologous boosters in later infancy. Dai et al. tested the immunogenicity of a heterologous prime-boost combination against TB in neonatal mice, with the initial DNA vaccine component given intradermally soon after birth followed by a recombinant adenovirus vector vaccine encoding the same antigen and given via the intranasal route at 6 weeks of age. Results suggested that neonatal immunisation with gene-based vaccines may create a favourable immunological environment that potentiates the pulmonary mucosal boosting, enhancing pulmonary T cell responses and therefore protection against pulmonary TB challenge [148, 149].

Combining the administration of BCG with other vaccines is already an established strategy which can enhance responses to co-delivered vaccines, as alluded to in the discussion on non-specific effects previously. Measurements of potential vaccine interference always form part of vaccine trials prior to the introduction of a new vaccine into WHO immunisation schedules; this principle needs to be preserved for any co-administered vaccines in the neonatal period with the appropriate longer term follow-up to discover any potential knock-on effects.

**Fig. 1:**
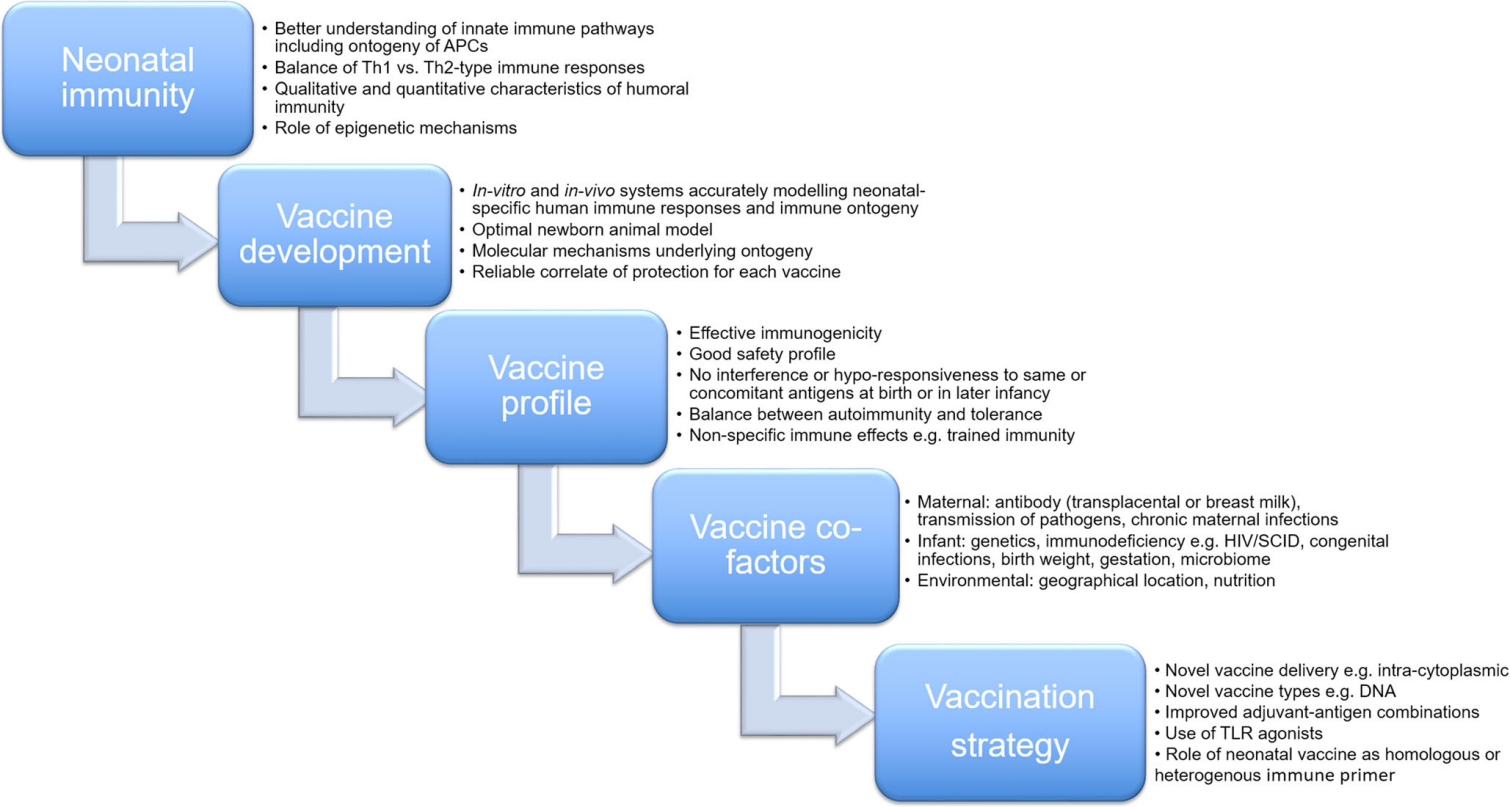
Summary of topics requiring further research to support of the development of future effective vaccines for use in newborns

Figure 1 summarises remaining gaps in knowledge and outlines research requirements for the development of the next generation of effective vaccines aimed at newborns.

## Conclusions

Effective neonatal vaccination remains a global health priority as it may have both immunological and practical advantages over other protective strategies in the newborn period. Three vaccines are currently administered successfully worldwide within the first 4 weeks of life: BCG, hepatitis B vaccine and oral polio vaccine. The difference in their mechanism of action, immune responses elicited and route of delivery effectively demonstrates the potential for impact of this type of immunisation on reducing morbidity and mortality of a range of possible neonatal and infant diseases. Newborn vaccines are also a useful and safe probe into neonatal immunity: by enabling the controlled delivery of a well-defined immune challenge to the naïve newborn immune system, we can develop a more comprehensive understanding of protective mechanisms in early life.

Potential barriers to successful neonatal vaccination are multi-factorial and include the following: inherent peculiarities of newborn innate and adaptive immunity limiting vaccine immunogenicity, intrinsic immunoregulatory mechanisms, host genetic factors, prematurity, infant co-morbidities, interference from maternal antibody, chronic maternal conditions and environmental influences.

Future research must integrate lessons from early immune ontogeny and focus on developing vaccine types with novel mechanisms of action that engage with the unique neonatal immune profile (Fig. 1). Optimising antigen-adjuvant combinations in order to activate APCs and potentiate innate immunity is key. This would promote secretion of IL-12, type I interferons and Th1-type polarising cytokines subsequently inducing robust Th1-type and TFH cell responses, thereby circumventing inherent deficiencies of the newborn adaptive immune system. Of note, a dual strategy involving newborn immune priming with subsequent homologous or heterologous boosting may be a particularly effective tool to tackle infections in early life. Rigorous and stringent safety analysis, however, must underpin all of these endeavours, including an accurate reporting system of both specific and non-specific generalised effects of novel vaccines.

**Table:** 

Key points
• Effective neonatal vaccination remains a global health priority with the potential to significantly reduce infant morbidity and mortality. • It is also a useful probing tool to improve our understanding of the protective innate and adaptive immune mechanisms in early life. • Only three vaccines are currently administered in the newborn period, BCG, hepatitis B vaccine and oral polio vaccine. • Recent advances in our understanding of immune ontogeny have resulted in a renewed interest in neonatal immunisation. • Other infant, maternal and environmental factors may have limiting effects on vaccine-induced immune responses. • Future strategies must overcome inherent deficiencies in neonatal immunity and address any concerns regarding vaccine immunogenicity, safety and immune interference.
